# The Effects of Hyperbaric Oxygen Therapy on Post-Training Recovery in Jiu-Jitsu Athletes

**DOI:** 10.1371/journal.pone.0150517

**Published:** 2016-03-09

**Authors:** Braulio Henrique Magnani Branco, David Hideyoshi Fukuda, Leonardo Vidal Andreato, Jonatas Ferreira da Silva Santos, João Victor Del Conti Esteves, Emerson Franchini

**Affiliations:** 1 Sports and Physical Education School of the University of Sao Paulo, Sao Paulo, Brazil; 2 Combat Sports and Martial Arts Research Group of the Physical Education and Sports School, University of Sao Paulo, Sao Paulo, Brazil; 3 University of Central Florida, Orlando, Florida, United States of America; 4 Santa Catarina State University, Florianopolis, Brazil; 5 Institute of Biomedical Sciences, University of Sao Paulo, Sao Paulo, Brazil; University of Alabama at Birmingham, UNITED STATES

## Abstract

**Objectives:**

The present study aimed to evaluate the effects of using hyperbaric oxygen therapy during post-training recovery in jiu-jitsu athletes.

**Methods:**

Eleven experienced Brazilian jiu-jitsu athletes were investigated during and following two training sessions of 1h30min. Using a cross-over design, the athletes were randomly assigned to passive recovery for 2 hours or to hyperbaric oxygen therapy (OHB) for the same duration. After a 7-day period, the interventions were reversed. Before, immediately after, post 2 hours and post 24 hours, blood samples were collected to examine hormone concentrations (cortisol and total testosterone) and cellular damage markers [creatine kinase (CK), aspartate aminotransferase (AST), alanine aminotransferase (ALT), and lactate dehydrogenase (LDH)]. Moreover, the rating of perceived exertion (RPE) and recovery (RPR) scales were applied.

**Results:**

Final lactate [La] values (control: 11.9 ± 1.4 mmol/L, OHB: 10.2 ± 1.4 mmol/L) and RPE [control: 14 (13–17 a.u.), OHB: 18 (17–20 a.u.)] were not significantly different following the training sessions. Furthermore, there was no difference between any time points for blood lactate and RPE in the two experimental conditions (P>0.05). There was no effect of experimental conditions on cortisol (F_1,20_ = 0.1, P = 0.793, η^2^ = 0.00, small), total testosterone (F_1,20_ = 0.03, P = 0.877, η^2^ = 0.00, small), CK (F_1,20_ = 0.1, P = 0.759, η^2^ = 0.01, small), AST (F_1,20_ = 0.1, P = 0.761, η^2^ = 0.01, small), ALT (F_1,20_ = 0.0, P = 0.845, η^2^ = 0.00, small) or LDH (F_1,20_ = 0.7, P = 0.413, η^2^ = 0.03, small). However, there was a difference between the two experimental conditions in RPR with higher values at post 2 h and 24 h in OHB when compared to the control condition (P<0.05).

**Conclusions:**

Thus, it can be concluded that OHB exerts no influence on the recovery of hormonal status or cellular damage markers. Nonetheless, greater perceived recovery, potentially due to the placebo effect, was evident following the OHB condition.

## Introduction

The recovery of athletes following training and during competition are a constant concern for technical staff since inadequate recovery can lead to fatigue, decreased performance, and increased potential for injury [1]. The ideal balance between training, competition, and physiological recovery are considered important factors in order to optimize performance [2]. However, there is little scientific evidence supporting the effective recovery of athletes by means of commonly used methods [3].

Typical Brazilian jiu-jitsu training sessions involve warm up, technical drills and combat simulation [4]. This type/method of training can cause muscle damage and may decrease competitive performance [5]. Additionally, physiological recovery between training sessions is pertinent to those athletes who participate in training cycles with a high number of competitions [6]. Recent studies indicate that the post-training use of cryotherapy results in the reduction of markers of muscle damage, such as creatine kinase (CK) and lactate dehydrogenase (LDH), while allowing for the maintenance of strength and a hypoalgesic effect in Brazilian jiu-jitsu athletes [5]. Similar results were obtained after a simulated Brazilian jiu-jitsu competition, where the findings indicated a significant reduction of CK, LDH and hypoalgesia among the athletes who participated in the study [5]. However, research carried out by Broatch et al. [7] with physically active individuals who were submitted to intensive training, established that the use of thermoneutral water obtained similar results to those found when using cryotherapy. This indicates that the physiological benefits (improved classification for pain threshold and readiness for exercise) demonstrated by cold water immersion may be related to the placebo effect. Within the context of these findings, evaluation of the various recovery modalities currently being utilized is needed.

Hyperbaric oxygen therapy is a treatment where 100% oxygen is administered under pressure greater than one absolute atmosphere (ATA). The process is based on pressurizing the blood vessels between 1.5–3.0 ATA for 60–120 min periods, once or twice a day [8]. The primary function of hyperbaric oxygen therapy is to accelerate the recovery of soft tissue by means of reducing local hypoxia, inflammation and edema [9,10,11]. Hyperbaric oxygen therapy has become a recommended form of treatment for recovery from injuries among non-athletes [12,13,14]. However, there is no scientific evidence that the method is effective in the treatment of elite athletes after training sessions and competition [2].

As such, the present study aims to examine the effect of hyperbaric oxygen therapy after intensive training sessions using markers of muscular damage and perceived recovery/exertion among Brazilian jiu-jitsu athletes. It is hypothesized that treatment with 100% oxygen will favor the recovery of markers of anabolic/catabolic status and muscular damage as well as the perception of recovery among participating athletes.

## Materials and Methods

### Subjects

The study included 11 experienced male adult Brazilian jiu-jitsu athletes (age: 29.7 ± 6.6 years old, body mass: 84.6 ± 15.8 kg, height: 179 ± 6 cm) with 8 ± 7 years of regular and systematic practice (5 blue-belts, 1 purple-belt, 3 brown-belts and 2 black-belts). The following inclusion criteria were required of participants: a 3-year minimum period of regular training (to ensure an adequate level of experience among athletes); participation in official competitions in the six months prior to the study; and a minimum Brazilian jiu-jitsu training frequency of three times per week (typical duration of 1.5 h per session). Athletes who were injured, in a process of weight loss or used illicit drugs (i.e., anabolic steroids), antibiotics and/or anti-inflammatory drugs were not accepted for the study. Athletes were requested to consume dinner on the evening prior to the training and breakfast one hour before training. The athletes were allowed to freely hydrate with water throughout the training sessions. In addition, the athletes were instructed not to take dietary supplements before or during data collection. The data collection was carried out on Mondays and blood samples were collected before training by a team of nurses. Training started at 11 am in both conditions. The athletes were instructed not to perform any type of vigorous physical exertion over the weekend, thus allowing for a 48-hour period of rest. Sample-size calculations indicated that 10 athletes would be necessary to detect a statistical difference based an effect size of 0.5, a 1-β error probability of 0.8 and a P < 0.05 for the main dependent variables. Thus, 12 athletes they were recruited according to the previous study by Shimoda et al. [15]. However, one athlete was excluded from the study due to injury during testing. This research was conducted in accordance with International Ethical Guidelines and Declaration of Helsinki, and was approved by Hospital Celso Ramos and State University of Santa Catarina, Florianopolis, Santa Catarina, Brazil approval number 4530021.1.00005360. The participants provided written informed consent to participate.

### Experimental design

**Fig 1:**
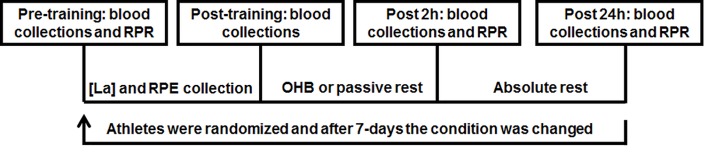
Experimental design. Note: [La] = Blood Lactate; RPE = Rating of perceived exertion (Borg 6–20); RPR = Rating of perceived recovery (0–10); OHB = hyperbaric oxygen therapy.

This study utilized a randomized cross-over design where Brazilian jiu-jitsu athletes were subjected to two experimental conditions following intensive training sessions. The experimental conditions included either passive recovery for 2 hours or hyperbaric oxygen therapy for a similar period of time. The actual duration of treatment in hyperbaric oxygen was 1 hour and 40 minutes, but approximately 20 minutes were allotted for the athletes to arrive at and enter the hyperbaric chamber. Prior to and throughout the recovery process (Fig 1), blood markers of anabolic/catabolic status and muscle damage were measured, while the perceived recovery of the athletes were recorded.

A wash-out period was utilized in order to avoid interference between the first and the second (carry-over) interventions. Subsequently, the alternate condition was undertaken for each participant [16]. Accordingly, a 7-day period was adopted between the two time-points with the intent of avoiding oscillations in the examined variables among participating athletes.

### Training sessions

The athletes carried out two typical Brazilian jiu-jitsu training sessions. Each session lasted for one hour and thirty minutes, comprised of a ten minute general and specific warm-up, technical training for twenty minutes (immobilizing, arm joint-locks and strangling) and six combat simulations of six minutes each, interspersed by four-minute periods of passive recovery. The combat simulations portion of the session was carried out with the highest possible intensity and the athletes received verbal encouragement from the coaching staff. In order to assess the intensity and assure that the sessions were similar, blood samples were collected from the ear lobe for blood lactate analysis at rest and after each combat simulation. Similarly, the rating of perceived exertion (RPE) scale [17] was applied at the same time-point as the blood lactate measurements (see below for further explanation).

### Hyperbaric oxygen therapy

The hyperbaric oxygen therapy was carried out by means of a multiplace Fogliene^®^ (model FH 2205) chamber with capacity for 14 people. The athletes were directed to the chamber, where they sat in individual chairs and oxygen was released through individual masks. The session started with a descent rate of approximately 7 meters per minute. The descent was interrupted at a depth of two meters (1.20 ATA) to allow for participants to put on their masks. From this moment, 100% oxygen concentration was released. At depths of five meters (1.50 ATA) and nine meters (1.90 ATA), the descent was interrupted in order to equalize internal ear pressure. Compression proceeded to a depth 13.9 meters (2.39 ATA), where it remained for 89 minutes. Thereafter, the depressurization process of the chamber was initiated at a rate of 4 meters per minute until a state of complete depressurization was reached. When the cycle ended, there was a treatment/recovery period (oxygen release) of one hour and forty minutes.

Pre-treatment glycemia (blood glucose: 112.7 ± 19.3 mg/dL) and blood pressure (systolic: 129 ± 8 mmHg and diastolic: 87 ± 9 mmHg) were evaluated for all athletes and values were within normal ranges after the training session (i.e., before going in to the hyperbaric chamber). A medical team was available throughout the whole treatment/recovery process to attend to participants, but no complications were diagnosed and no medical assistance was required.

### Blood samples and Biochemical analysis

Blood samples (15 ml) were collected from the antecubital veins at predetermined time points (Fig 1). The collections were performed through the use of Injex^®^ 20 ml syringes (Ourinhos, Brazil) and BD Vacutainer^®^ (Franklin Lates, United States of America) type Scalp 21 G disposable needles. After collection, 4 ml of blood were immediately dispensed into Petrodis^®^ (Sao Paulo, Brazil) 4 ml tubes with ethylenediamine tetra acetic acid (EDTA) to obtain the plasma. The remaining contents were dispensed in glass tubes without anticoagulant for serum extraction. Both tubes, with and without anticoagulant, were centrifuged at 3000 rpm for 15 minutes at 4°C, for separation into serum and plasma. Furthermore, intraclass correlation coefficient (ICC) values for the hormonal concentrations and cellular damage markers were calculated from the pre-training values using both experimental conditions.

To measure hormonal action in the plasma, an enzyme linked immunosorbent assay (ELISA) was used to determine cortisol and total testosterone concentrations with the use of Arbor^®^ (Michigan, United States of America) commercial kits. The ICC for cortisol was 0.71 and 0.91 for total testosterone.

For markers of cell damage, lactate dehydrogenase (LDH) was measured in serum by the kinetic method, and creatinine levels by the colorimetric method. Within the plasma, creatine kinase (CK) was measured by the kinetic method and aspartate aminotransferase (AST) and alanine aminotransferase (ALT) levels were measured by the colorimetric method. Analyses were performed with the use of Gold Analisa^®^ (Belo Horizonte, Brazil) commercial kits and a Bioplus^®^ UV-2000 (Sao Paulo, Brazil) spectrophotometer. The ICC for CK was 0.90, 0.87 for LDH, 0.79 for AST and 0.57 for ALT.

To measure blood lactate concentration, blood samples (25 μL) were collected at the earlobe with heparinized capillaries and were immediately stored in microtubes containing 50 μL of sodium fluoride at 1% for analysis using a Yellow Springs YSI 1500^®^ lactimeter (Ohio, United States of America). Moreover, two solutions were prepared containing 50 μL sodium fluoride at 1% and 25 μL of standard solution of 5 mmol/L as a standardization method for homogenizing the blood samples according to the environment in which data collection was performed. Furthermore, ICCs for blood lactate were calculated throughout the training sessions, with the following results: pre-training (0.89) and after each-fight (1st: 0.74; 2nd: 0.86; 3rd: 0.78; 4th: 0.99; 5th: 0.67 and 6th: 0.97).

### Rating of Perceived Exertion and Perceived Recovery Status

After the training session, the athletes were questioned about their RPE based on the 6–20 scale (10). The ICCs for RPE throughout the training sessions were calculated, with the following results: pre-training (0.81) and after each-fight (1st: 0.94; 2nd: 0.91; 3rd: 0.90; 4th: 0.86; 5th: 0.90 and 6th: 0.94). In order to verify the rating of perceived recovery (RPR) of athletes, the scale proposed by Laurent et al. [18] was used, with scores ranging from 0 (very poorly recovered/extremely tired) to 10 (recovered well/highly rested), before, two hours after and 24 hours after training in both conditions (control and experimental). Moreover, the pre-training ICC for RPR was 0.81.

### Statistical Analysis

The parametric data are presented as mean and standard deviation. The Kolmogorov-Smirnov test was conducted to determine normality and the Levene test was utilized to determine homogeneity of the data. A comparison across the different time points was performed using two-way (treatment and time) analysis of variance (ANOVA) with repeated measures, followed by a Bonferroni post hoc test. A Mauchly’s test of sphericity was used to test this assumption and a Greenhouse-Geisser correction was applied when necessary. All interactions (condition×time) are described in the tables. In the text, interactions are reported only for the same time-point in different conditions. Additionally, to evaluate the magnitude of the observed differences, the effect size was calculated (*eta squared*, η^2^) and interpreted as follows: < 0.2 (small), > 0.2 and < 0.8 (moderate) and > 0.8 (large) [19]. For the ordinal data, RPE and RPR, non-parametric statistics were used in accordance with Bishop and Herron [20]. In order to compare conditions, the Wilcoxon test was used and data was represented by the median and percentiles (25% and 75%). Furthermore, the effect size for non-parametric data was calculated through the equation (*r* = *Z*/√*N*) wherein the r indicated the effect size and N is the number of observations. This values was interpreted in accordance with Field [21]: < 0.30 (small), > 0.30 and < 0.50 (medium) and > 0.50 (large), respectively. The significance level was set at P < 0.05. The data were analyzed using the Statistica software version 12.0 (Stasoft, United States of America) and the SPSS software version 20.0 (IBM, United States of America).

## Results

Table 1 presents the lactate concentration and RPE among Brazilian jiu-jitsu athletes during the control and hyperbaric oxygen therapy conditions.

**Table 1:** Lactate concentration and rating of perceived exertion (RPE) in Brazilian jiu-jitsu athletes at rest and following the combat simulations for the control and hyperbaric oxygen therapy conditions (n = 11).

	Control		Hyperbaric Oxygen Therapy	
Fight simulation	Lactate (mmol/L)^a^	RPE (6–20 a.u.)^b^	Lactate (mmol/L)^a^	RPE (6–20 a.u.)^b^
Rest	0.9 ± 0.5*	6 (6–7)	0.9 ± 0.4*	7 (6–7)
Post 1	8.5 ± 1.1^†^	12 (9–13)	9.5 ± 1.5^§^	15 (10–15)
Post 2	9.9 ± 1.4 ^‡^	14 (12–15)	10.0 ± 1.5	14 (10–15)
Post 3	10.3 ± 1.6	15 (13–16)	11.6 ± 1.6	15 (10–17)
Post 4	10.5 ± 1.3	15 (13–17)	10.6 ± 1.4	14 (12–16)
Post 5	10.9 ± 1.7	15 (13–15)	11.4 ± 1.8	15 (14–18)
Post 6	11.9 ± 1.4	14 (13–17)	10.2 ± 1.4	18 (17–20)

Note: The two experimental conditions showed similar physiological perturbations among participants

a = data are expressed as mean ± standard deviation

b = data are expressed as median and percentiles (25% and 75%); a.u. = arbitrary unit

* = different from the post-fight 1, 2, 3, 4, 5, and 6 (P < 0.001) for lactate in both conditions

† = different from post-fight 3, 4, 5 and 6 (P < 0.05) in same condition

‡ = different from post-fight 6 in the same condition (P < 0.01)

§ = different from post-fight 3 and 5 in the same condition (P < 0.05); There was no between-group differences at any time-point for RPE (P > 0.05).

For blood lactate, there was a time effect (F_6,120_ = 225.04; P < 0.001, η^2^ = 0.918, large), with resting values being lower than any of the post-fight values (P < 0.01). There was also an interaction effect (F_6,120_ = 3.89; P = 0.001, η^2^ = 0.163, small) where resting blood lactate concentrations were different from all post-fight values for both groups (P < 0.001), but not between conditions for any of the time-points (P > 0.05) (Table 1).

For RPE, there was no difference between the two experimental conditions at the at any of the selected time-points (P > 0.05) (Table 1).

Table 2 presents the RPR in Brazilian jiu-jitsu athletes during the control and hyperbaric oxygen therapy conditions.

**Table 2:** Rating of perceived recovery (RPR) in Brazilian jiu-jitsu athletes prior to and following training during for the control and hyperbaric oxygen therapy conditions (n = 11).

	Control–RPR (0–10 a.u.)	Hyperbaric Oxygen Therapy–RPR (0–10 a.u.)
Pre	10 (9–10)	9 (9–10)
Post 2h*	6 (5–7)	8 (6–9)
Post 24h*	9 (8–9)	10 (9–10)

Note: data are expressed as median and percentiles (25% and 75%); a.u. = arbitrary unit

* = higher values from experimental condition when compared to control (P < 0.05).

For RPR, there was a between-condition difference at post 2 h (Z = 2.52, P = 0.012; r = 0.76, large) and post 24 h (Z = 2.37, P = 0.018; r = 0.71, large), with higher values from hyperbaric oxygen therapy when compared to the control condition (Table 2).

Table 3 presents the hormonal and cellular damage responses in Brazilian jiu-jitsu athletes during the control and hyperbaric oxygen therapy conditions.

**Table 3:** Hormonal responses and cellular damage prior to and following training in Brazilian jiu-jitsu athletes for the control and hyperbaric oxygen therapy conditions (n = 11).

	Control				Hyperbaric Oxygen Therapy			
	Pre	Post	Post 2h	Post 24h	Pre	Post	Post 2h	Post 24h
Cortisol (ng/ml) *^,^**	10.4 ± 2.6	18.1 ± 4.7	8.1 ± 1.8	15.6 ± 4.1	11.6 ± 3.2	19.3 ± 4.6	6.5 ± 2.0	15.6 ± 3.5
Testosterone (ng/ml) ^‡^	449 ± 195	429 ± 137	316 ± 116	467 ± 149	435 ± 129	435 ± 144	314 ± 138	441 ± 152
CK (U/L) *^,^ **	365 ± 144	503 ± 179	511 ± 163	562 ± 335	327 ± 132	508 ± 270	502 ± 322	489 ± 281
AST (U/L) *^,^ **	28 ± 6	36 ± 7	33 ± 8	33 ± 8	27 ± 4	35 ± 6	33 ± 6	33 ± 8
ALT (U/L) *^,^ **^,^ ^ǂ^^,^ ^#^	36 ± 11	45 ± 15	43 ± 16	44 ± 14	41 ± 9	46 ± 10	42 ± 10	42 ± 9
LDH (U/L) **	204 ± 33	243 ± 48	244 ± 55	199 ± 39	186 ± 31	230 ± 35	221 ± 41	197 ± 47

Note: data are expressed as mean ± standard deviation; CK = creatine kinase; AST = aspartate aminotransferase; ALT = alanine aminotransferase; LDH = lactate dehydrogenase

* = difference in pre-training and both post-training and post 24 h between experimental conditions (P < 0.001)

** = difference in post 2 h and pre-training for both experimental conditions (P < 0.05)

‡ = difference in post 2 hours and all time points for both experimental conditions (P < 0.001)

ǂ = difference in the pre-training between experimental conditions (P < 0.001)

# = difference in post 24 h when compared with post-training and post 2 h between experimental conditions (P < 0.05)

There were no condition or interaction effects for any of the hormonal concentrations or cellular damage markers (P > 0.05) (Table 3).

For cortisol, there was a time effect (F_3,60_ = 52.6, P < 0.001, η^2^ = 0.72, moderate), with higher values post-training and post 24 h, and lower values post 2 h when compared to pre-training values (P < 0.01). Furthermore, the values post-training were higher when compared to 2 h and 24 h post-training (P < 0.05) and post 2 h values were lower when compared to post 24 h values (P < 0.001).For total testosterone, there was a time effect (F_3,60_ = 15.4, P < 0.001, η^2^ = 0.43, moderate), with higher values in pre, post-training and post 24 h when compared to measurements performed post 2 h (P < 0.001) (Table 3).

For CK, there was a time effect (F = 10.5, P < 0.001, η^2^ = 0.35, moderate), with higher values in all moments when compared to pre-training values (P < 0.001) (Table 3).

For AST, there was a time effect (F_3,60_ = 22.4, P < 0.001, η^2^ = 0.53, moderate), with higher values in all conditions when compared with pre-training (P < 0.001), and high value when compared to post-training values (P = 0.03) (Table 3).

For ALT, there was a time effect (F_3,60_ = 8.8, P < 0.001, η^2^ = 0.31, moderate), with higher values in all moments when compared with pre-training (P < 0.05) (Table 3).

For LDH, there was a time effect (F_3,60_ = 33.6, P < 0.001, η^2^ = 0.63, moderate), with higher values post-training and post 2 h (P < 0.001) when compared to pre-training values and higher values post-training and post 2 h when compared to post 24 h (P < 0.001) (Table 3).

## Discussion

The main goals of this study were to investigate the effect of post-training hyperbaric oxygen therapy on physiological, perceptive and hormone responses in experienced Brazilian jiu-jitsu athletes. The blood lactate and RPE values were similar in the two conditions (experimental and control conditions) across time-points, indicating similar efforts and intensities during the two training sessions. No significant differences in physiological or hormonal responses were found between the control and hyperbaric oxygen therapy conditions. However, post-training RPR values (at 2 h and 24 h) were greater following acute hyperbaric oxygen therapy when compared to the control condition. The currently reported improvement in RPR following hyperbaric oxygen therapy appears to be in agreement with the results of Kim et al. [22] in physically active men showing decreased fatigue perception after 40 min exposure to 1.3 ATA. However, with the altered RPR being the only significant difference between conditions, the potential impact of the placebo effect, as demonstrated by Broatch et al. [7][6] in response to cold water immersion following repeated sprints, must be considered. Despite no concomitant changes in hormone concentrations and cellular damage markers, the current findings with regard to RPR must considered within the context of competitive athletes. Accordingly, the classification of RPR, as proposed by Laurent et al. [18], may be an important indicator of readiness and performance throughout a training cycle.

While the hyperbaric oxygen therapy intervention did not modify the cell damage markers or the hormone concentrations (cortisol and total testosterone), the results of this study indicate that the typical Brazilian jiu-jitsu training sessions increased CK, AST, and ALT concentrations immediately post-training, post 2 h and 24 h for both conditions. Likewise, the two training sessions increased LDH concentrations immediately post-training and post 2 h, but returned to resting levels after 24 h. Hence, the adoption of strategies for physiological recovery may be relevant to maintain performance in Brazilian jiu-jitsu athletes, especially during competitive periods [6].

The treatment of injuries arising from athletic training and competition by means of hyperbaric oxygen therapy has increased exponentially over the past three decades [23]. Notwithstanding, the benefits of hyperbaric oxygen therapy for treatment of sports injuries may be limited, because the location of the injury seems to influence the effectiveness of the treatment. Previous reports have noted that muscle injuries appear less likely to benefit from hyperbaric oxygen therapy for the purpose of recovery, than areas of reduced perfusion, such as tendons and ligaments [24]. Nonetheless, there are no studies in the literature that have assessed the physiological recovery from athletic training, or combat sports, by means of hyperbaric oxygen therapy.

Furthermore, Babul et al. [25] did not detect a hyperbaric oxygen therapy-related reduction in muscle damage markers in sedentary college-aged women following execution of 300 eccentric maximal contractions (30 sets of 10 repetitions per minute) using an isokinetic dynamometer. In contrast, a recent study reported that acute treatment by means of hyperbaric oxygen therapy was able to reduce muscle fatigue during the execution of plantar flexion exercises in physically active subjects [15]. Notably, the aforementioned study utilized a monoarticular exercise with fixed resistance. This does not reflect the physiological and metabolic demands of Brazilian jiu-jitsu training and competition, which are characterized by multi-joint movements performed at high-intensities with opponents of varying technical and physical fitness profiles [26].

In relation to hormone responses, elevated cortisol levels immediately post-training and post 24 h were observed when compared to resting and post 2 h values for the two experimental conditions. Concerning total testosterone, lower values post 2 h were observed when compared to all other time points (pre, post and post 24 h). In turn, no data were found in the extant literature regarding the effect of hyperbaric oxygen therapy on the hormonal response to combat sports, or sporting activities in general. However, Passavanti et al. [27] showed elevated testosterone concentrations after several hyperbaric oxygen therapy sessions (ranging from 4 to 23 sessions) in a patients with pathologic conditions and healthy controls. The above-mentioned study did not require physical effort and patients were subjected to a chronic protocol rather than a single treatment session as examined in the current investigation. Nonetheless, increased serum testosterone, a potential marker of anabolism and an indicator of recovery status, may occur following multiple hyperbaric oxygen therapy sessions which could be beneficial for Brazilian jiu-jitsu athletes.

Conversely, cortisol has been utilized as a marker of catabolism [28]. A previous study showed that 60 min exposure at 2.5 ATA with 100% oxygen was able to reduce cortisol concentrations after intervention [29]. However, this study evaluated divers, who had distinct characteristics and training adaptations as compared to Brazilian jiu-jitsu athletes. Brazilian jiu-jitsu training requires great neuromuscular demand (aerobic power, grip endurance and muscle power to carry out the guard passing techniques, sweeps, takedowns, back control and submissions) as well as moderate activation of the glycolytic pathway within intermittent activities (high and low intensities) [26]. Cortisol plays an important role during physical effort, acting as a counter-regulator to maintain the glycemic levels of athletes [30]. The physical effort demanded in the present study was likely higher than in the activities carried out by divers, who are familiarized with the pressurization process, both of which are factors that could have contributed to differences in cortisol response. Cortisol secretion is also influenced by circadian rhythm and may further explain discrepancies following hyperbaric oxygen therapy. Data collection in the current study started at 11:00 am, whereas Lund et al. [29] conducted their diving investigation from 8:00 am. Moreover, the current results appear to be in conflict with those reported in an animal model, which showed an increase of circulating cortisol concentrations after exposure by means of hyperbaric oxygen therapy [31]. Due to the lack of research, studies seeking to investigate the mechanisms triggered by hyperbaric oxygen therapy must be conducted to improve our understanding of this type of intervention.

We can regard the absence of post-training (2 and 24 h) performance tests in both experimental conditions as one of the limitations of the present study. Furthermore, the inclusion of a third condition in the study, in which the athletes were situated in the hyperbaric chamber inhaling ambient oxygen through the individual masks would have allowed for a more thorough examination of the placebo effect. This condition was not feasible due to technical limitations of the hyperbaric chamber to provide ambient oxygen during pressuration which would have minimized the effectiveness of a placebo condition. Finally, the combat simulation protocol presented in this investigation yielded highly reproducible data as evident by high ICC values across training conditions for almost all of the analyzed variables.

## Conclusion

In summary, the currently investigated method of hyperbaric oxygen therapy, utilized during physiological recovery in Brazilian jiu-jitsu athletes, was not effective. As a consequence of these findings, hyperbaric oxygen therapy using altered protocols, such as increasing the session time and exposure, conducting two sessions in the same day, or chronic post-training exposure over the course of a training cycle, should be examined. In addition, future studies should evaluate the effects of this recovery modality on performance and consider including a placebo condition.
